# Identification of a Major Locus for Lodging Resistance to Typhoons Using QTL Analysis in Rice

**DOI:** 10.3390/plants12030449

**Published:** 2023-01-18

**Authors:** Dan-Dan Zhao, Yoon-Hee Jang, Eun-Gyeong Kim, Jae-Ryoung Park, Rahmatullah Jan, Lubna, Sajjad Asaf, Saleem Asif, Muhammad Farooq, Hyunjung Chung, Dong-Jin Kang, Kyung-Min Kim

**Affiliations:** 1 Department of Applied Biosciences, Kyungpook National University, Daegu 41566, Republic of Korea; 2 Crop Foundation Research Division, National Institute of Crop Science, Rural Development Administration, Wanju 55365, Republic of Korea; 3 Crop Breeding Division, National Institute of Crop Science, Rural Development Administration, Wanju 55365, Republic of Korea; 4 Coastal Agriculture Research Institute, Kyungpook National University, Daegu 41566, Republic of Korea; 5 Natural and Medical Science Research Center, University of Nizwa, Nizwa 616, Oman; 6 Teaching and Research Center for Bio-Coexistence, Faculty of Agriculture and Life Science, Hirosaki University, Gosyogawara 037-0202, Japan

**Keywords:** quantitative trait locus, lodging resistance, typhoon, cytochrome P450, rice breeding, climate change

## Abstract

We detected a new target quantitative trait locus (QTL) for lodging resistance in rice by analyzing lodging resistance to typhoons (Maysak and Haishen) using a scale from 0 (no prostrating) to 1 (little prostrating or prostrating) to record the resistance score in a Cheongcheong/Nagdong double haploid rice population. Five quantitative trait loci for lodging resistance to typhoons were detected. Among them, *qTyM6* and *qTyH6* exhibited crucial effects of locus RM3343–RM20318 on chromosome 6, which overlaps with our previous rice lodging studies for the loci *qPSLSA6-2*, *qPSLSB6-5*, and *qLTI6-2*. Within the target locus RM3343–RM20318, 12 related genes belonging to the cytochrome P450 protein family were screened through annotation. *Os06g0599200* (*OsTyM/Hq6*) was selected for further analysis. We observed that the culm and panicle lengths were positively correlated with lodging resistance to typhoons. However, the yield was negatively correlated with lodging resistance to typhoons. The findings of this study improve an understanding of rice breeding, particularly the culm length, early maturing, and heavy panicle varieties, and the mechanisms by which the plant’s architecture can resist natural disasters such as typhoons to ensure food safety. These results also provide the insight that lodging resistance in rice may be associated with major traits such as panicle length, culm length, tiller number, and heading date, and thereby improvements in these traits can increase lodging resistance to typhoons. Moreover, rice breeding should focus on maintaining suitable varieties that can withstand the adverse effects of climate change in the future and provide better food security.

## 1. Introduction

The frequency and intensity of natural disasters are increasing under the influence of climate change, causing severe damage to crop production and threatening food security [1]. The importance of adapting to climate change has been described previously [2]. Rice is a vital food crop, and its stable production substantially affects global politics and economics [3]. Lodging is a major limiting factor in rice production worldwide [4]. Lodging reduces yield and quality by decreasing photosynthesis in the canopy, disrupts vascular bundles by bending or breaking stalks, and causes difficulties in mechanical harvesting [5]. Strong winds bend the rice culm and rainstorms flood farmland, causing rice grains and roots to rot after long-term soaking and seriously affecting rice production [6]. Rice losses caused by typhoons in Japan [7,8] and Taiwan [9] have been reported.

Conventional breeding techniques combined with recent advances in biotechnology and genomics, such as the use of quantitative trait locus (QTL) analysis for genetic analysis of complex traits, are helpful to better understanding the genetic basis of lodging resistance in rice. Plant height is a quantitative trait controlled by QTLs and plays an important role in lodging. For plant height, a major QTL is *qph8*, detected on chromosome 8 between the marker interval RM502-RM264 using the recombinant inbred lines [10]. Likewise, there are five QTLs for controlling the pushing strength of the lower stem, and *prl5* from Kasalath on chromosome 5 had a positive effect echoed by lodging resistance to the typhoon [11].

Consequently, it is necessary to analyze rice plants’ lodging during typhoons and to breed rice varieties with high breaking strengths to endure typhoons of rising intensity; it is especially vital to identify QTLs related to strong culms. In 2010, Ookawa et al. identified, through QTLs which improve culm strength, a superior allele of STRONG CULM 1 and 2 (SCM1 and SCM2) in the *indica* variety Habataki [12]. Similarly, in a tropical *japonica* variety called Chugoku 117, identical STRONG CULM 3 and 4 (SCM3 and SCM4) QTLs were discovered [13].

In rice breeding, reducing plant height has historically been the main goal for improving lodging resistance [11,14]. Plant height is closely related to lodging resistance at different developmental stages of cereals [15]. Numerous recent reports documented the isolation of plant height-related genes. For example, the heterotrimeric G protein is a dwarf gene involved in gibberellin acid (GA) signalling that was cloned into rice [16,17]. Approximately 70 dwarf-associated mutants were reported in rice, many of which have been described as GA-deficient or gibberellin-insensitive [18]. The phytohormone GA is involved in many plant developmental processes, including shoot elongation and plant height [19]. Additionally, Ishimaru et al., 2008 identified a target for rice lodging resistance (*lrt5*) and its role in typhoons through QTL analysis [20]. Moreover, the number of days from the panicle and maturity period was significantly negatively correlated with lodging, whereas stem diameter, stem length, weight, and length of panicle were significantly positively correlated with lodging resistance in rice [21]. Therefore, analyzing rice plants lodged during typhoons is crucial. In an earlier study of QTL for lodging resistance to typhoons using Nipponbare and Kasalath BILs, the results showed that two QTLs were detected on chromosome 5 and one QTL was detected on chromosome 6, the nearest marker locus being R2549 [11].

In this study, we analysed lodging resistance to typhoons in rice using a Cheongcheong/Nagdong double haploid (CNDH) population and its recipient parent Cheongcheong/Nagdong to identify the QTL controlling lodging resistance to typhoons in the CNDH population. Our results may facilitate the breeding of rice varieties resistant to lodging to overcome the adverse effects of climate change.

## 2. Results

### 2.1. Phenotype Evaluation for Lodging Resistance to Typhoons and Other Agricultural Traits

**Figure 1:**
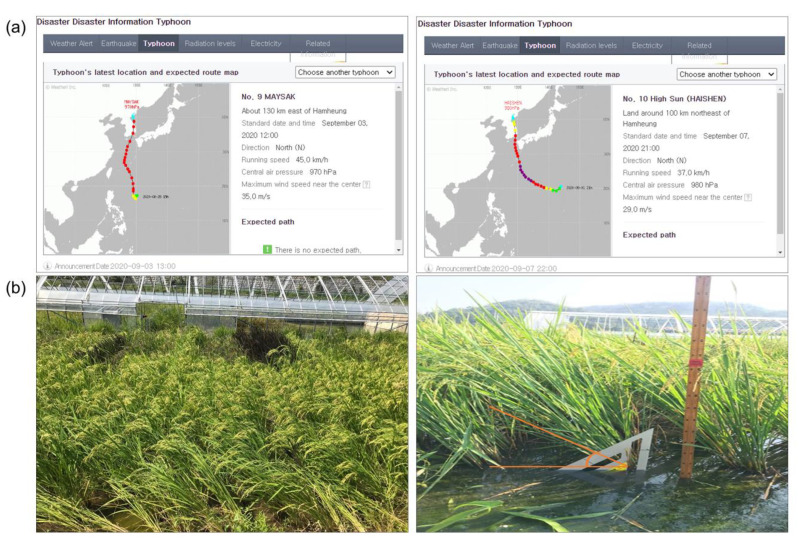
Information on typhoons Maysak and Haishen, and observation of plants during these periods. (**a**) Lodging resistance to typhoons was observed on 4 September 2020, the day after No. 9 Typhoon Maysak, and 8 September 2020, the day after No. 10 Typhoon Haishen. Typhoon Maysak had a running speed of 45 km/h, a central air pressure of 970 hPa, and a maximum wind speed near the centre of 35.0 m/s. Typhoon Haishen had a running speed of 37.0 km/h, a central air pressure of 980 hPa, and a maximum wind speed near the centre of 29.0 m/s. (**b**) Resistance rating was recorded on a scale of 0 (no prostrating) to 1 (little prostrating or prostrating).

**Figure 2:**
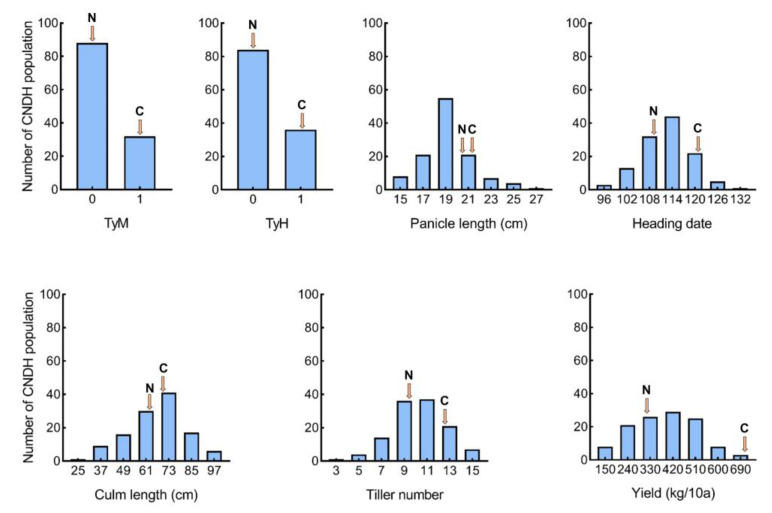
Frequency distribution of phenotypic values of lodging resistance to typhoons in the CNDH population (TyM, Typhoon Maysak; TyH: Typhoon Haishen; C, Cheongcheong; N, Nagdong).

Based on data collected after Typhoons Maysak and Haishen (Figure 1) for lodging resistance to typhoons, Cheongcheong buckled completely, whereas Nagdong remained straight. After Typhoon Maysak, 88 lines had no prostration, whereas 32 lines had little prostration or prostration in the CNDH population. For the CNDH population after Typhoon Haishen, 84 lines remained straight, and 36 were lodged. The results revealed that, after experiencing two severe typhoons, lodging became aggravated. In addition, the observed traits (panicle length, heading date, culm length, tiller number, and yield) exhibited a similar frequency distribution curve as that of the normal distribution in the CNDH population (Figure 2). The agricultural traits measured are quantitative traits controlled by polygenes. The average culm and panicle lengths of Cheongcheong were longer than those of Nagdong (Table S1). In the CNDH population, the mean culm and panicle lengths were 66.19 cm and 19.50 cm, respectively. The tiller number and yield of Cheongcheong were higher than those of Nagdong. Furthermore, Nagdong has a shorter heading date compared with that of Cheongcheong. According to correlation analysis, typhoons Maysak and Haishen had a negative correlation with yield and a positive correlation with the panicle and culm lengths in 2020. However, the tiller number and heading date were not correlated with lodging resistance to typhoons. Moreover, the panicle and culm length show a positive correlation with each other and with the heading date, while the tiller number shows a negative correlation with culm length (Table S2). 

### 2.2. QTL Analysis Associated with Lodging Resistance to Typhoons

**Figure 3:**
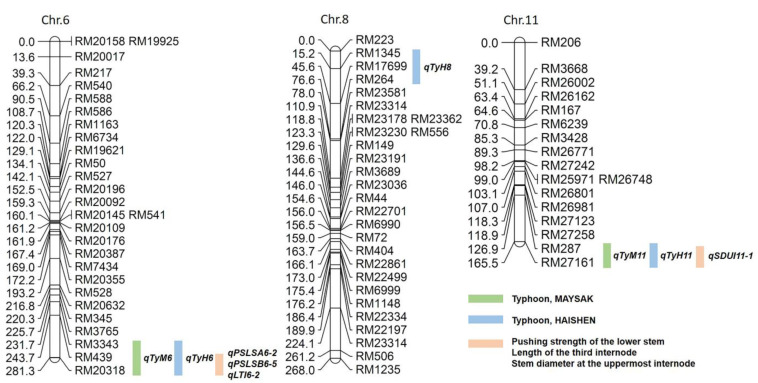
The location of QTLs on chromosomes associated with lodging resistance to typhoons in the CNDH population. QTL analysis suggests that genes related to lodging resistance to typhoons are located on chromosomes 6, 8, and 11.

**Figure 4:**
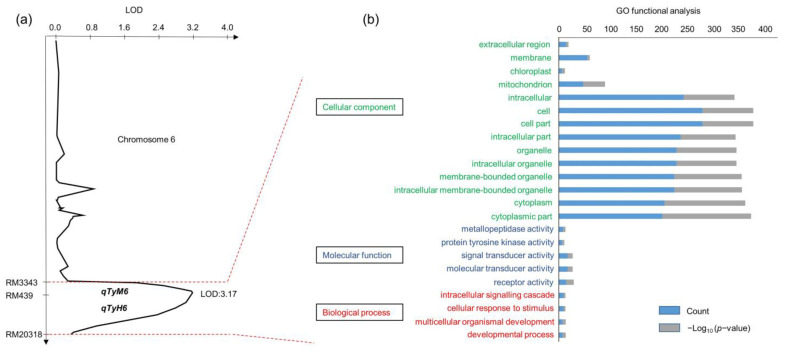
The LOD score and Gene Ontology (GO) annotation of the target QTLs are between RM3343 and RM20318. (**a**) LOD score of the QTL for resistance to lodging during typhoon Maysak and Haishen on chromosome 6 (*qTyM6* and *qTYH6*). (**b**) GO functional analysis of genes related to the target locus between RM3343 and RM20318.

Lodging resistance to typhoons Maysak and Haishen was detected using QTL analysis. Following the two typhoons, five QTLs on chromosomes 6, 8, and 11 were detected (Figure 3). All QTLs had positive alleles in Cheongcheong. Among them, on chromosome 6, the loci *qTyM6* and *qTyH6* overlapped in the QTL interval flanked by markers RM3343 and RM20318. In addition, the loci *qTyM11* and *qTyH11* were detected in the QTL interval flanked by markers RM287 and RM27161. On chromosome 8, *qTyH8* detected markers between RM1345 and RM264 (Table S3). The QTL of lodging resistance to typhoons explained 32–51% of phenotypic variance in the CNDH population. The logarithm of odds (LOD) was between 2.55–3.59. Here, the LOD scores of *qTyM6* and *qTyH6* were >3.0 (Figure 4a). Therefore, the target marker interval RM3343–RM20318 on chromosome 6 was predicted as the major control locus.

### 2.3. Gene Exploration from Target QTL Marker Interval RM3343–RM20318

**Figure 5:**
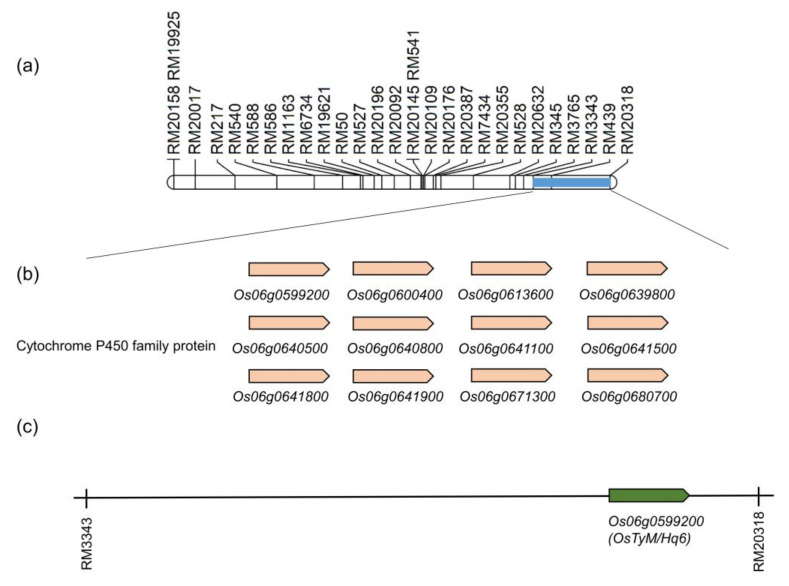
Physical mapping of genes related to lodging resistance to typhoons. (**a**) Target interval RM3343–RM20318 on chromosome 6. (**b**) 12 related genes belonging to cytochrome P450 family protein, selected as related genes between RM3343 and RM20318 on chromosome 6. (**c**) Screening of *OsTyM/Hq6*, among the related genes, as a target gene-related to lodging resistance to typhoons.

Genes related to lodging resistance to typhoons within the marker interval RM3343–RM20318 on chromosome 6 were filtered using the rice expression profile database and rice annotation project database. We screened 190 related genes in the target region RM3343–RM20318 using gene annotation (Table S4). Furthermore, the functional classification of genes within the marker interval RM3343–RM20318 on chromosome 6 in rice was predicted (Figure 4b) using the systems biology of AgriGO as a reference database. Significant Gene Ontology (GO) terms for marker interval RM3343–RM20318-related genes were identified, and 14, five, and four significant terms were identified in the ‘cellular component’, ‘molecular function’, and ‘biological process’ categories, respectively. The most enriched GO terms related to ‘cellular component’ were a response to cytoplasmic part, cytoplasm, and intracellular membrane-bounded organelle; enriched terms associated with ‘molecular function’ included receptor activity, molecular transducer activity, signal transducer activity, protein tyrosine kinase activity, and metallopeptidase activity; and enriched terms in ‘biological process’ were developmental process, multicellular organismal development, cellular response to stimulus, and intracellular signalling cascade. Moreover, 12 genes related to lodging resistance to typhoons belonging to the cytochrome P450 family proteins were selected according to existing sequence annotations, with *Os06g0599200*, named as *OsTyM/Hq6*, selected as a target gene (Figure 5).

### 2.4. Sequence Analysis of OsTyM/Hq6

**Figure 6:**
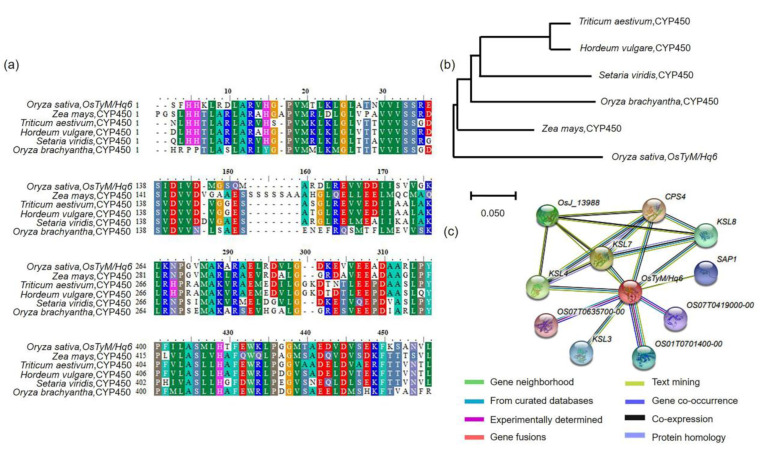
Sequence analysis of *OsTyM/Hq6*. (**a**) Comparison of the protein sequences of *OsTyM/Hq6* homologous genes; substantially high similarity was found in *Zea mays*, *Hordeum vulgare*, *Setaria viridis*, *Triticum aestivum*, and *Oryza brachyantha*. (**b**) The phylogenetic tree was used to analyze *OsTyM/Hq6* and its homologous genes. The phylogenetic tree was constructed with 1000 bootstrap replicates using the parsimony method. (**c**) Protein interaction of *OsTyM/Hq6*. The gene interacts with OsJ_13988, CPS4, KSL4, KSL7, OS07T0635700-00, SAP1, OS07T0419000-00, KSL3, and OS01T0701400-00.

The target *OsTyM/Hq6* was selected for lodging resistance to typhoons using QTL mapping in 120 CNDH populations. In addition, BLAST analysis using the NCBI database indicated that *OsTyM/Hq6* has a highly similar sequence to cytochrome P450 from *Zea mays*, *Triticum aestivum*, *Hordeum vulgare*, *Setaria viridis*, and *Oryza brachyantha* (Figure 6a). Phylogenetic tree analysis confirmed the genetic similarity of *OsTyM/Hq6* in *Z. mays*, *T. aestivum*, *H. vulgare*, *S. viridis*, and *O. brachyantha* (Figure 6b). Furthermore, using the domains of *OsTyM/Hq6* to predict functional partners, *OsTyM/Hq6* was found to interact with 10 different proteins (OsJ_13988, CPS4, KSL8, KSL7, KSL4, OS07T0635700-00, OS07T0419000-00, KSL3, OS01T0701400-00, and SAP1) (Figure 6c).

## 3. Discussion

Lodging resistance is a vital trait necessary for achieving high yields in rice production [22]. Recent developments in QTL analysis technology and molecular marker mapping have increased the accuracy of QTL analysis of rice lodging resistance [12,21,23,24]. None of the previous studies overlapped with our results, indicating that the CNDH population we bred over the past decade provided a large genetic resource for improving lodging resistance in rice. The CNDH population has become a bridge parent with various characteristics. Therefore, in the present study we detected new loci, *qTyM6* and *qTyH6*, involved in lodging resistance to typhoons. Furthermore, we previously evaluated the pushing strength of the lower stem and internode length to detect the QTL involved in lodging resistance [25,26] in the CNDH population. The results overlapped with similar loci (*qPSLSA6-2*, *qPSLSB6-5*, *qLTI6-2*, and *qSDUI11-1*) on chromosomes 6 and 11 (Figure 3 and Table S5). These results indicate that reducing plant height can improve lodging resistance by improving the pushing strength of the lower stem in rice. Moreover, lodging resistance to typhoons can be enhanced by introducing the loci *qTyM6* and *qTyH6* to reduce plant height and lower plant thrust resistance. The lodging index previously measured, of pushing strength of the lower stem before and after heading using a digital force gauge and the internode length, was consistent with the typhoon-induced lodging index in nature, as these methods detected similar loci. Furthermore, in field experiments we used a planting distance of 30 × 15 cm; during this field condition 70% of the lines remained straight (84 lines with no prostrating and 36 lines with little prostrating or prostrating), proving that 30 × 15 cm is a suitable planting distance to resist lodging caused by typhoons. The loci *qTyM6* and *qTyH6* are flanked by the interval markers RM3343 and RM20318, which contain 190 related genes to lodging resistance. According to gene annotation analysis, 12 cytochrome P450 genes were identified. 

In plants, cytochrome P450s account for approximately 1% of protein-coding sequences. They are the largest family of enzymes involved in plant metabolism, including GA biosynthesis, catabolism, and the synthesis of primary and secondary metabolites [27,28,29]. As a phytohormone, GA is involved in regulating numerous vital growth and developmental processes in rice [30,31]. In addition, among the proteins and genes involved in plant growth, development, and adaptation to biotic and abiotic stresses, plant cytochrome P450 monooxygenases are one of the largest families [32]. Previous research suggested that increased GA accumulation in plants with mutant *EUI1* (encoding a putative cytochrome P450 monooxygenase) led to abnormal elongation of the uppermost internodes [33]. Moreover, two other *CYP714* gene family members encode GA13 oxidases (*CYP714B1* and *CYP714B2*) that negatively regulate rice shoot development and are involved in GA homeostasis [34]. In the present study, the significant target marker interval RM3343–RM20318 contained 12 cytochrome P450 genes. Among these genes, we focused on *Os06g0599200* (*OsTyM/Hq6*), which belongs to the cytochrome P450 family. As cytochrome P450 is involved in the GA biosynthesis pathway, it may be a useful aspect to generate a semi-dwarf rice cultivar with a more productive and higher stem diameter. The nucleotide sequence of *OsTyM/Hq6* is the same as those found in *Z. mays*, *T. aesivum*, *H. vulgare*, *S. viridis*, and *O. brachyantha*. *Dwarf3* encodes cytochrome P450, which mediates an early regulatory step in GA biosynthesis in *Z. mays* [35]. Furthermore, *OsTyM/Hq6* interacted with the proteins OsJ_13988 (involved in momilactone phytoalexin biosynthesis), CPS4 (syn-copalyl diphosphate synthase), KSL4 (involved in momilactone phytoalexin biosynthesis), KSL7 (involved in phytocassane phytoalexin biosynthesis), SAP1 (involved in environmental stress response), KSL3 (ent-kaurene synthase-like 3), OS07T0635700-00, OS01T0701400-00, and OS07T0419000-00 (belonging to the cytochrome P450 family).

In this study, our results show that lodging resistance to typhoons can be modified by the amplification of *qTyM6* and *qTyH6*, as our correlation results showed that the panicle and culm lengths positively correlated with lodging resistance to typhoons Maysak and Haishen. It was confirmed that the reduction of plant height is the target for genetic improvement of lodging resistance in rice. Moreover, the QTL analysis of lodging resistance to typhoons detected the target marker interval RM3343-RM20318, having 12 repeated genes belonging to cytochrome P450 genes, which are involved in GA biosynthesis and affect plant height. Mutant plants with cytochrome P450 enzyme may play vital roles in rice plants by generating semi-dwarf rice that can overcome lodging resistance with more significant yield and productivity. This provided a significant approach for improving the rice’s lodging resistance by the introduction of the cytochrome P450 gene; in the next step, we intend to analyze the mechanism of the cytochrome P450 gene for lodging resistance, in particular the target gene *OsTyM/Hq6*.

## 4. Materials and Methods

### 4.1. Field Experiment Design and Plant Material

We used 120 CNDH populations and their parental lines to analyze lodging resistance to typhoons and other agricultural traits. Experiments were conducted in 2020 in a rice field (Gunwi, Gyeongbuk, Republic of Korea, latitude 36°11′, east longitude 128°64′). Seed surfaces were disinfected with 25% prochloraz (Hankook Samgong, Seoul, Republic of Korea) and then soaked in tap water for three days at 33 °C in an incubator. The parent lines (Cheongcheong and Nagdong) and 120 CNDH population were transplanted at a density of 0.3 × 0.15 m, with all populations randomized into a block design. Field management was performed according to the rules of the Rural Development Administration in Korea, with K_2_O-P_2_O_5_-N at a 5.7:4.5:9 kg/10a ratio used as fertilizer. 

### 4.2. Mapping Population

A genetic map was constructed from anther cultures of the varieties mentioned earlier [36]. The CNDH population map was constructed using 788 simple-sequence repeat (SSR) markers. Polymorphisms were analysed using 423 SSR markers, among which 222 SSR markers were screened by PCR amplification of co-dominant genes [37]. The total genetic distance of the CNDH population genetic map was 2121.7 cM, and the average genetic distance between markers was 10.6 cM [36]. The CNDH genetic map was drawn using Mapmaker 3.0, and the markers were evenly distributed on the 12 rice chromosomes [38].

### 4.3. Measurement of Lodging Resistance to Typhoons and Other Agricultural Traits

Important agricultural traits associated with rice lodging, such as lodging resistance to typhoons, heading date, panicle length, culm length, tiller number, and yield, were evaluated. Lodging resistance to typhoons was observed on 4 September 2020, which was the day after No. 9 Typhoon Maysak, and 8 September 2020, which was the day after No. 10 Typhoon Haishen. The running speed of Typhoon Maysak was 45 km/h, its central air pressure was 970 hPa, and its maximum wind speed near the centre was 35.0 m/s. Typhoon Haishen’s running speed was 37.0 km/h, its central air pressure was 980 hPa, and its maximum wind speed near the centre was 29.0 m/s (https://www.weather.go.kr/plus/typ/report.jsp (accessed on 10 September 2022). We used a scale from 0 (no prostrating) to 1 (little prostrating or prostrating) to record the resistance score of the CNDH population [11]. The plant culm length was estimated from the bottom of the paddy field to the panicle neck, and the panicle length was measured from the panicle neck to the grain end. The tiller number of each plant was measured for both effective and non-effective tillers.

### 4.4. QTL and Statistical Analyses of Lodging Resistance to Typhoons and Other Agricultural Traits

QTLs related to lodging resistance to typhoons and other agricultural characteristics were analyzed using Windows QTL Mapper 2.5 [39]. We used the nomenclature proposed by McCouch and Doerge to name the QTLs detected [40]. The Windows QTL Mapping program version 2.5 requires several factors, such as labels for all markers, the genetic distance between each marker, genotyping data, the number of chromosomes, and the value of the trait of interest. Composite interval mapping was performed for the entire genome. Moreover, the threshold of the logarithm of odds (2.5) was used for all data. Six replicates of the average measurements of agricultural traits were used for follow-up analysis. Statistical analyses were performed by calculating the mean and standard deviation. Analysis and drawing of frequency distribution graphs were performed using GraphPad Prism software (version 8.0.2; GraphPad, Inc., La Jolla, CA, USA). Pearson’s correlation analysis was performed using SPSS software (version 28.0.1.1; SPSS, Inc., Chicago, IL, USA).

### 4.5. Prediction of Related Genes and Gene Information Analysis

Analysis of lodging-resistance-related genes is valuable for addressing deficiencies in QTL analysis. To physically map and annotate associated genes, we used the rice annotation project database [41] and the rice expression profile database [42]. Open reading frames were found among the SSR markers, and functional classification of related genes was performed according to the gene annotations. The AgriGO tool was used for GO enrichment analysis to determine the functions of related genes (http://bioinfo.cau.edu.cn/agriGO/ (accessed on 7 November 2022) [43]. The National Center for Biotechnology Information website [44] and BioEdit 7.0 [45] were used to compare multiple homologous sequences. The protein–protein interaction/association network was analyzed using the STRING (version 11.0) (https://string-db.org/ (accessed on 15 November 2022) database.

## 5. Conclusions

We performed QTL analysis of rice plants’ lodging resistance to typhoons Maysak and Haishen, which occurred in 2020. The target interval between markers RM3343 and RM20318 on chromosome 6 was determined. *OsTyM/Hq6* was selected from the 12 cytochrome P450 genes, as it appeared most frequently among the 190 related genes. The major target genes and QTLs involved in lodging resistance to typhoons can be used in breeding programs to develop environmentally appropriate varieties for climate change. The molecular properties of *OsTyM/Hq6* should be further evaluated. Studies are also required to determine the molecular mechanism of this gene and rice lodging resistance. The data collected during natural typhoons are similar to those collected by researchers using machines, such as the pushing resistance of the lower stem and internode length. Thus, researchers can perform rice breeding based on data obtained using current technology to overcome the adverse effects of climate change.

## Data Availability

Not applicable.

## Supplementary Materials

The following supporting information can be downloaded at: https://www.mdpi.com/article/10.3390/plants12030449/s1, Table S1: Phenotypic and lodging resistance to typhoon values in the CNDH population and its parents, Cheongcheong and Nagdong; Table S2: Correlation of phenotypic and lodging resistance to typhoon values from the 120 CNDH populations in 2020; Table S3: QTLs related to lodging resistance to typhoons of the CNDH population in 2020; Table S4: Related gene list in the interval flanked by markers RM3343 and RM20318 for lodging resistance to typhoons using QTL analysis; Table S5: Comparison of the QTLs for lodging resistance to typhoons with our previous studies using the CNDH population.
