# Supplementary material for: Identification of a Major Locus for Lodging Resistance to Typhoons Using QTL Analysis in Rice
Source: Plants (Basel). 2023 Jan 18;12(3):449. doi: 10.3390/plants12030449 (PMC9919122; doi:10.3390/plants12030449)
Supplement: Supplementary file 1 [file plants-12-00449-s001.zip › plants-2086899-supplementary.pdf]

Table S1. Phenotypic and lodging resistance to typhoon values in the CNDH population and its parents, Cheongcheong and Nagdong.

Trait	Parents		DH Population		
	**Cheongcheong**	**Nagdong**	**Max.**	**Min.**	**Mean**
TyM	1.00	0.00	1.00	0.00	0.27 ± 0.44
TyH	1.00	0.00	1.00	0.00	0.30 ± 0.46
Heading date	123	110	129	96	112.03 ± 6.90
Panicle length	21.50 ± 0.70	20.60 ± 2.20	27.00	13.00	19.50 ± 2.50
Culm length	68.00 ± 4.56	65.40 ± 4.67	101.10	29.60	66.19 ± 15.30
Tiller number	12.70 ± 0.50	9.70 ± 0.70	17.70	3.30	10.95 ± 2.46
Yield	712.20	317.10	670.32	153.55	394.90 ± 123.73

TyM: Typhoon Maysak. TyH: Typhoon Haishen

Table S2. Correlation of phenotypic and lodging resistance to typhoon values from the 120 CNDH populations in 2020.

	**TyM**	**TyH**	**Culm length**	**Panicle length**	**Heading date**	**Tiller number**	**Yield**
TyM	1.000						
TyH	0.921**	1.000					
Culm length	0.345**	0.386**	1.000				
Panicle length	0.363**	0.381**	0.735**	1.000			
Heading date	-0.079	-0.095	0.356**	0.290**	1.000		
Tiller number	0.005	-0.021	-0.188*	-0.078	-0.098	1.000	
Yield	-0.367**	-0.377**	0.038	0.031	0.154	-0.028	1.000

TyM: Typhoon Maysak. TyH: Typhoon Haishen

** Significant at 0.01 level

* Significant at the 0.05 level

Table S3. QTLs related to lodging resistance to typhoons of the CNDH population in 2020.

Characteristics	Locus	Chromosome	Interval Markers [a]	LOD	Add. Effect [b]	R^2 [c]	Increasing effects [d]
Lodging resistance by Typhoons	*qTyM6*	6	RM3343–RM20318	3.08	0.14	0.32	Cheongcheong
	qTyM11	11	RM287–RM27161	2.55	0.19	0.40	Cheongcheong
	qTyH6	6	RM3343–RM20318	3.17	0.14	0.38	Cheongcheong
	qTyH8	8	RM1345–RM264	2.77	0.19	0.45	Cheongcheong
	qTyH11	11	RM287–RM27161	3.59	0.22	0.51	Cheongcheong

TyM: Typhoon Maysak. TyH: Typhoon Haishen

[a] Marker intervals are those within the significance threshold on each border of the QTL range.

[b] Additive effect.

[c] Phenotypic variation explains each QTL.

[d] Increase effect is the source of the allele causing an increase in the measured traits.

Table S4. The related genes list of the interval markers RM3343-RM20318 for lodging resistance to typhoons using QTL analysis.

Locus ID	Description
Os06g0594100	Enoyl-CoA hydratase/isomerase domain containing protein.
Os06g0594600	Transferase family protein.
Os06g0595700	Transposase, IS4 domain containing protein.
Os06g0595800	Transferase family protein.
Os06g0595900	Transcription elongation factor S-II, central region domain containing protein.
Os06g0597500	Protein prenyltransferase domain containing protein.
Os06g0597600	Aromatic-ring hydroxylase family protein.
Os06g0597800	FAR1 domain containing protein.
Os06g0597900	SOUL heme-binding protein family protein.
Os06g0599200	Cytochrome P450 family protein.
Os06g0600400	Cytochrome P450 family protein.
Os06g0600700	Agenet domain containing protein.
Os06g0601500	cAMP response element binding (CREB) protein family protein.
Os06g0602600	Alba, DNA/RNA-binding protein family protein.
Os06g0602800	Glycosyl transferase, family 14 protein.
Os06g0602900	Alcohol dehydrogenase superfamily, zinc-containing protein.
Os06g0604200	Phospholipase D.
Os06g0604300	Phospholipase D.
Os06g0604500	Mitochondrial substrate carrier family protein.
Os06g0605100	Zinc finger, BED-type predicted domain containing protein.
Os06g0605900	Leucine-rich repeat, cysteine-containing subtype containing protein.
Os06g0606800	Targeting for Xklp2 family protein.
Os06g0607200	Cellular retinaldehyde-binding/triple function, C-terminal domain containing protein.
Os06g0607700	ABC transporter related domain containing protein.
Os06g0607900	No pollen.

Os06g0608500	Snf7 family protein.
Os06g0608800	von Willebrand factor, type A domain containing protein.
Os06g0609600	EF-Hand type domain containing protein.
Os06g0609700	Esterase/lipase/thioesterase domain containing protein.
Os06g0610800	Peptidase A1, pepsin family protein.
Os06g0611200	Protein prenyltransferase domain containing protein.
Os06g0611400	Virulence factor, pectin lyase fold family protein.
Os06g0612200	RNA polymerase Rpb1, domain 5 containing protein.
Os06g0613100	Protein prenyltransferase domain containing protein.
Os06g0613600	Cytochrome P450 family protein.
Os06g0614000	C2 calcium/lipid-binding region, CaLB domain containing protein.
Os06g0614100	Eukaryotic transcription factor, DNA-binding domain containing protein.
Os06g0614300	Cyclase-associated protein domain containing protein.
Os06g0617800	Ribose-phosphate pyrophosphokinase 2
Os06g0618100	Zinc finger, CCCH-type domain containing protein.
Os06g0618700	VQ domain containing protein.
Os06g0619000	Disease resistance protein family protein.
Os06g0620600	Hrf1 family protein.
Os06g0621500	Disease resistance protein family protein.
Os06g0621600	Disease resistance protein family protein.
Os06g0622300	AT-rich interaction region domain containing protein.
Os06g0622700	Eukaryotic transcription factor, DNA-binding domain containing protein.
Os06g0623300	NAD-dependent epimerase/dehydratase family protein.
Os06g0624100	Proteinase inhibitor, propeptide domain containing protein.
Os06g0625200	Peptidoglycan-binding LysM domain containing protein.
Os06g0625300	Peptidoglycan-binding LysM domain containing protein.
Os06g0625900	Potassium transporter 10 (OsHAK10).
Os06g0626600	Sulfotransferase family protein.

Os06g0626700	Isopenicillin N synthase family protein.
Os06g0627500	Leucine-rich repeat, plant specific containing protein.
Os06g0633300	Phytosulfokines 1 precursor
Os06g0633500	Zinc finger, RING-type domain containing protein.
Os06g0633800	Amino acid/polyamine transporter II family protein.
Os06g0633900	Esterase/lipase/thioesterase domain containing protein.
Os06g0634100	Intron maturase, type II family protein.
Os06g0636700	Lipolytic enzyme, G-D-S-L family protein.
Os06g0638000	Zinc finger, CCCH-type domain containing protein.
Os06g0638300	Retrotransposon gag protein family protein.
Os06g0638500	Protein kinase domain containing protein.
Os06g0639200	Pathogenesis-related transcriptional factor and ERF domain containing protein.
Os06g0639300	Ankyrin repeat containing protein.
Os06g0639800	Cytochrome P450 family protein.
Os06g0640100	E-class P450, group I family protein.
Os06g0640500	Cytochrome P450 family protein.
Os06g0640800	Cytochrome P450 family protein.
Os06g0641100	Cytochrome P450 family protein.
Os06g0641500	Cytochrome P450 family protein.
Os06g0641800	Cytochrome P450 family protein.
Os06g0641900	Cytochrome P450 family protein.
Os06g0642900	Ubiquitin system component Cue domain containing protein.
Os06g0643100	Proteasome subunit beta type 3
Os06g0643600	Formamidopyrimidine-DNA glycolase family protein.
Os06g0643700	Hly-III related proteins family protein.
Os06g0644100	Carboxypeptidase regulatory region domain containing protein.
Os06g0644300	Disease resistance protein family protein.
Os06g0644500	Zinc finger, DHHC-type domain containing protein.

Os06g0644700	Amino acid/polyamine transporter II family protein.
Os06g0644800	Mannose-6-phosphate receptor, binding domain containing protein.
Os06g0645400	Aminoacyl-tRNA synthetase, class 1a, anticodon-binding domain containing protein.
Os06g0645500	Sterile alpha motif homology domain containing protein.
Os06g0645600	Peptidase S1 and S6, chymotrypsin/Hap domain containing protein.
Os06g0646000	SAM (and some other nucleotide) binding motif domain containing protein.
Os06g0646400	Protein kinase domain containing protein.
Os06g0646600	KNOX family class 2 homeodomain protein.
Os06g0649700	Region of unknown function DM13, Skeletor domain containing protein.
Os06g0649800	DNA glycosylase family protein.
Os06g0649900	Phospholipase D/Transphosphatidylase domain containing protein.
Os06g0650600	Nonaspanin (TM9SF) family protein.
Os06g0650900	Heat shock protein DnaJ family protein.
Os06g0651300	Zinc finger, C2H2-type domain containing protein.
Os06g0652200	Hly-III related proteins family protein.
Os06g0653100	CD9/CD37/CD63 antigen family protein.
Os06g0653200	Basic helix-loop-helix dimerisation region bHLH domain containing protein.
Os06g0654000	Peptidase C19, ubiquitin carboxyl-terminal hydrolase 2 family protein.
Os06g0654300	Histidine kinase, homodimeric domain containing protein.
Os06g0655200	Pollen allergen Lol p2 family protein.
Os06g0656800	Beta-Ig-H3/fasciclin domain containing protein.
Os06g0658900	UbiA prenyltransferase family protein.
Os06g0659200	Intron maturase, type II family protein.
Os06g0660800	Protein kinase domain containing protein.
Os06g0661000	Plant MuDR transposase domain containing protein.
Os06g0661400	ANTH domain containing protein.
Os06g0661600	Zinc finger, DHP-type domain containing protein.
Os06g0661700	RabGAP/TBC domain containing protein.

Os06g0662200	Eukaryotic transcription factor, DNA-binding domain containing protein.
Os06g0662300	Pollen allergen Lol p2 family protein.
Os06g0662500	Pollen allergen Lol p2 family protein.
Os06g0662600	Pollen allergen Lol p2 family protein.
Os06g0662700	Pollen allergen Lol p2 family protein.
Os06g0662800	Pollen allergen Lol p2 family protein.
Os06g0662900	Pollen allergen/expansin, C-terminal domain containing protein.
Os06g0663300	Pollen allergen Lol p2 family protein.
Os06g0663500	SBP domain containing protein.
Os06g0663600	Endonuclease V family protein.
Os06g0664200	Inositol phosphatase/fructose-1,6-bisphosphatase family protein.
Os06g0664400	HMG-I and HMG-Y, DNA-binding domain containing protein.
Os06g0665500	Amidase, hydantoinase/carbamoylase family protein.
Os06g0665800	ATPase, P-type, K/Mg/Cd/Cu/Zn/Na/Ca/Na/H-transporter family protein.
Os06g0665900	Thioredoxin domain 2 containing protein.
Os06g0666400	VQ domain containing protein.
Os06g0667200	MADS30.
Os06g0669800	Ovarian tumour, otubain domain containing protein.
Os06g0671300	Cytochrome P450 family protein.
Os06g0671800	Cellular retinaldehyde binding/alpha-tocopherol transport family protein.
Os06g0677000	Patatin family protein.
Os06g0677300	RINGv domain containing protein.
Os06g0677400	Hydroxyacid dehydrogenase/reductase family protein.
Os06g0677500	Protein prenyltransferase domain containing protein.
Os06g0679100	Cdc23 domain containing protein.
Os06g0679800	Heat shock protein Hsp70 family protein.
Os06g0680700	Cytochrome P450 family protein.
Os06g0681200	Cupredoxin domain containing protein.

Os06g0681300	PAK-box/P21-Rho-binding domain containing protein.
Os06g0681400	Polyubiquitin.
Os06g0681600	Haem peroxidase family protein.
Os06g0682800	Zinc finger, CCCH-type domain containing protein.
Os06g0682900	(2R)-phospho-3-sulfolactate synthase, ComA family protein.
Os06g0683000	Zinc finger, C2H2-type domain containing protein.
Os06g0683100	NAD-dependent epimerase/dehydratase family protein.
Os06g0683500	Ribonuclease H domain containing protein.
Os06g0685300	C2 domain containing protein.
Os06g0686400	Plant lipid transfer protein/Par allergen family protein.
Os06g0686500	Peptidase M3A and M3B, thimet/oligopeptidase F domain containing protein.
Os06g0687100	Ras GTPase family protein.
Os06g0687200	Zinc finger, RING-type domain containing protein.
Os06g0687500	RNA-binding region RNP-1 (RNA recognition motif) domain containing protein.
Os06g0687900	Glycosyl transferase, family 43 protein.
Os06g0688100	RNA polymerase I specific transcription initiation factor RRN3 family protein.
Os06g0690200	Protein kinase domain containing protein.
Os06g0690900	Protein prenyltransferase domain containing protein.
Os06g0691000	DNA repair protein, Rev1 family protein.
Os06g0691100	Pathogenesis-related transcriptional factor and ERF domain containing protein.
Os06g0691600	EF-Hand type domain containing protein.
Os06g0692700	Leucine rich repeat, N-terminal domain containing protein.
Os06g0693100	Disease resistance protein family protein.
Os06g0693500	Zinc finger, C2H2-type domain containing protein.
Os06g0694200	Lipolytic enzyme, G-D-S-L family protein.
Os06g0695300	Haem peroxidase, plant/fungal/bacterial family protein.
Os06g0695400	Haem peroxidase family protein.
Os06g0695600	Zinc finger, RING-type domain containing protein.

Os06g0695700	Calmodulin-binding, plant family protein.
Os06g0695800	ABC transporter related domain containing protein.
Os06g0695900	Zinc finger, RING-type domain containing protein.
Os06g0696900	Appr-1-p processing domain containing protein.
Os06g0697200	DEAD/DEAH box helicase domain containing protein.
Os06g0697400	RNA polymerase Rpb8 family protein.
Os06g0697500	AAA ATPase domain containing protein.
Os06g0697600	AAA ATPase domain containing protein.
Os06g0698300	Protein phosphatase 2C family protein.
Os06g0698400	RNA-binding region RNP-1 (RNA recognition motif) domain containing protein.
Os06g0698600	Exo70 exocyst complex subunit family protein.
Os06g0698900	Zinc finger, GATA-type domain containing protein.
Os06g0699400	MAP kinase 2.
Os06g0699500	Macrophage migration inhibitory factor family protein.
Os06g0699600	CCT domain containing protein.
Os06g0700000	Proteinase inhibitor, propeptide domain containing protein.
Os06g0700100	Protein prenyltransferase domain containing protein.
Os06g0701100	Eukaryotic initiation factor 4A (eIF4A) (eIF-4A).
Os06g0701200	UTP--glucose-1-phosphate uridylyltransferase family protein.
Os06g0701300	Beta-lactamase family protein.
Os06g0701600	Cation transporter family protein.
Os06g0701700	HKT-type transporter (Sodium ion transporter).
Os06g0701900	Auxin responsive SAUR protein family protein.
Os06g0702000	Auxin responsive SAUR protein family protein.
Os06g0702100	Zinc finger, CW-type domain containing protein.

Table S5. Comparison of the QTLs for lodging resistance to typhoons with our previous studies using the CNDH population.

Characteristics	Locus	Chromosome	Interval Markers	LOD
Lodging resistance to typhoons	*qTyM6*	6	RM3343–RM20318	3.08
	qTyM11	11	RM287–RM27161	2.55
	qTyH6	6	RM3343–RM20318	3.17
	qTyH11	11	RM287–RM27161	3.59
Pushing strength of the lower stem	*qPSLSA6-2*	6	RM439–RM20318	3.23
	qPSLSB6-5	6	RM439–RM20318	3.13
Length of the third internode	*qLTI6-2*	6	RM439–RM20318	3.65
Stem diameter at the uppermost internode	*qSDUI11-1*	11	RM287–RM27161	2.56

TyM: Typhoon Maysak. TyH: Typhoon Haishen. PSLSA: pushing strength of the lower stem after the heading date. PSLSB: pushing strength of the lower stem before the heading date. LTI: length of the third internode. SDUI: stem diameter at the uppermost internode.
